# Digital Engagement in Healthcare: The Influence of Internet Use and Social Media on Messaging Providers, Viewing Test Results, and Scheduling Appointments

**DOI:** 10.3390/healthcare14193358

**Published:** 2026-10-08

**Authors:** Banee Louis, Gulzar Shah, Bettye Apenteng, Jeffery Jones

**Affiliations:** Department of Health Policy and Management, Jiann-Ping Hsu College of Public Health, Georgia Southern University, Statesboro, GA 30460, USAbapenteng@georgiasouthern.edu (B.A.); jajones@georgiasouthern.edu (J.J.)

**Keywords:** digital health, online health services, Internet use, digital engagement, appointment scheduling, healthcare provider communication

## Abstract

**Background/Objectives**: Digital platforms are increasingly used to access health information and healthcare services, yet engagement with online health services varies across individuals. This study examined factors associated with three forms of digital health engagement among U.S. adults: using the Internet to message healthcare providers or their offices, view medical test results, and make medical appointments. **Methods**: Cross-sectional data from the 2022 Health Information National Trends Survey (HINTS 6) were analyzed. Survey-weighted multivariable logistic regression models were used to examine associations between the three digital health engagement outcomes and Internet use for health information, social media use for health decision-making, confidence in finding helpful health resources online, trust in the healthcare system, and delayed needed care, while adjusting for selected demographic and health characteristics. **Results**: Across all three models, respondents who used the Internet to look for health information had substantially higher odds of messaging healthcare providers (AOR = 9.88, 95% CI [7.59, 12.87]), viewing test results (AOR = 14.46, 95% CI [10.80, 19.37]), and making medical appointments online (AOR = 7.38, 95% CI [5.16, 10.54]). Lower confidence in finding helpful health resources online was also associated with lower odds of selected forms of digital health engagement. Delayed needed care was associated with lower odds of viewing test results, while age and race/ethnicity were associated with selected forms of digital engagement. **Conclusions**: Internet health information seeking and confidence in finding helpful health resources online were consistently associated with engagement in online health services. These findings highlight the importance of prior online health experience and confidence in understanding patterns of digital healthcare engagement and may inform efforts to promote equitable access to and use of online healthcare services.

## 1. Introduction

Digital healthcare has evolved beyond the digitization of medical records to include a broad range of technologies that support access to health information, communication, and healthcare delivery [1,2]. The Internet, in particular, has become an important resource for individuals seeking health information and interacting with healthcare providers. Patients can increasingly use online services to communicate with providers, view medical test results, and schedule appointments. These functions may support patient engagement and facilitate access to healthcare services. However, engagement with digital health services is not uniform. Understanding the factors associated with the use of these services is increasingly important as healthcare systems continue to expand digital modes of communication and access.

Despite the growing availability of digital health services, disparities in their use persist. Previous research suggests that demographic and socioeconomic characteristics such as age, education, and income can influence engagement with digital health tools [3,4]. Other factors like chronic health conditions, privacy concerns, system usability, perceived usefulness, and accessibility have also been noted as influencing factors [5,6]. Confidence in navigating online health resources may also shape whether individuals use digital healthcare services. As digital interactions become increasingly integrated into healthcare delivery, identifying factors associated with engagement is important for understanding who may benefit from these services and who may encounter barriers to their use.

Prior studies have examined several factors associated with digital health engagement. However, less is known about how health information-seeking behaviors, confidence in finding helpful health resources online, and social media use for health decision-making are associated with specific forms of online healthcare engagement. The roles of trust in the healthcare system and delayed needed care are also less understood. Examining these factors together may provide a better understanding of how individuals engage with online provider communication and healthcare services.

Trust may also play a role in individuals’ engagement with digital healthcare. Recent research using HINTS 6 data has examined the relationship between trust in healthcare and the use of digital health technologies [7]. Other research has identified multiple dimensions of trust within digital health systems [8]. However, trust in digital health technologies or information is conceptually distinct from broader trust in the healthcare system. Because online services such as provider messaging, test-result access, and appointment scheduling are increasingly integrated into healthcare delivery, examining healthcare-system trust may provide additional insight into individuals’ engagement with these services. Accordingly, this study examines whether trust in the healthcare system is associated with the three forms of digital health engagement considered here.

Social media has become an important source of health information and can influence health-related attitudes, decisions, and behaviors [9]. However, the increasing use of social media for health information has also raised concerns about exposure to health misinformation [10]. While previous research has examined the role of social media in health-related behaviors, its relationship with engagement in online healthcare services remains less clear. In this study, social media use refers specifically to the use of information from social media to make health decisions rather than direct communication with healthcare providers through social media platforms. Healthcare access may also be associated with digital engagement. Individuals may delay needed medical care because of barriers such as cost, transportation, and access to healthcare providers [11]. However, less is known about whether delayed needed care is associated with engagement in online healthcare services. This study therefore examines the relationship between delayed needed care and three forms of digital health engagement: messaging healthcare providers, viewing medical test results, and making medical appointments online. The present study extends prior research by examining multiple forms of online healthcare engagement within the same nationally representative analysis and by considering health-information seeking, confidence in finding helpful online health resources, social media use for health decision-making, healthcare-system trust, and delayed needed care simultaneously. Examining these factors across distinct forms of digital engagement may provide a more comprehensive understanding of how individuals interact with online healthcare services.

This study examines factors associated with three forms of digital health engagement among U.S. adults: (1) using the Internet to message a healthcare provider or provider’s office, (2) viewing medical test results online, and (3) making medical appointments online. Specifically, associations between these behaviors and Internet use for health information, use of social media information for health decision-making, confidence in finding helpful health resources online, trust in the healthcare system, and delayed needed care, while accounting for chronic conditions, biological sex, education, age, and race/ethnicity, were examined. By examining these distinct forms of engagement, this study seeks to identify factors associated with how individuals interact with online healthcare services and to inform efforts to reduce barriers to digital health engagement.

The Unified Theory of Acceptance and Use of Technology (UTAUT) provides a conceptual framework for considering factors that may shape individuals’ engagement with digital technologies. UTAUT identifies four core determinants of technology acceptance and use: performance expectancy, effort expectancy, social influence, and facilitating conditions [12]. In the present study, UTAUT is used as a theory-informed framework to organize factors potentially associated with digital health engagement rather than as a formal test of the UTAUT model. Internet health-information seeking and confidence in finding helpful health resources online represent aspects of individuals’ experience and confidence with digital resources, while the use of social media information for health decision-making reflects a potential social influence on health-related behavior. Trust in the healthcare system, chronic health conditions, and delayed needed care provide additional healthcare-context factors that may be associated with digital engagement. Because the HINTS 6 measures used in this study were not designed as validated measures of UTAUT constructs, they are not treated as direct operationalizations of the original UTAUT dimensions.

## 2. Materials and Methods

### 2.1. Study Design and Data Source

This study used a cross-sectional quantitative design to examine factors associated with three forms of digital health engagement: using the Internet to message a healthcare provider or provider’s office, view medical test results, and make medical appointments online. The cross-sectional data from the Health Information National Trends Survey (HINTS 6), conducted by the National Cancer Institute in 2022, were used. HINTS 6 is a nationally representative survey that collects data on Americans’ knowledge, attitudes, and behaviors related to health information, with a focus on cancer prevention and detection. The survey used a dual-mode approach: postal delivery and push-to-web invitations for online completion. To account for potential biases, the data are weighted to provide nationally representative estimates.

### 2.2. Study Sample and Inclusion/Exclusion Criteria

The initial HINTS 6 sample included 6252 respondents aged 18 years or older, of whom 6185 completed the survey, and 67 provided partial responses. For the present study, responses categorized as “Not Reported” for the three digital health engagement outcome variables—using the Internet to message healthcare providers, view medical test results, and make medical appointments—were excluded to retain binary outcome measures, resulting in a descriptive analytic sample of 5983 respondents. For the multivariable regression analyses, respondents who reported that they did not need medical care during the previous 12 months were excluded from the delayed-needed-care measure to avoid ambiguity in interpreting this variable. Three observations with unreported Internet health-information-seeking status were also excluded. The final regression analytic sample included 4675 respondents. For the remaining independent variables, missing or inapplicable responses were retained as a separate “Not Reported” category to minimize additional loss of observations. The “Not Reported” responses were treated as categorical indicators in the analyses rather than as numeric values. These categories were retained to minimize additional loss of observations and should be interpreted primarily as indicators of missing or inapplicable responses rather than as substantively meaningful respondent groups. Accordingly, estimates for “Not Reported” categories were not interpreted substantively, even when statistically significant.

Inclusion criteria:

- Adults aged 18 years or older who responded to HINTS 6 (2022)
- Valid responses to all three digital health engagement outcome variables (messaging healthcare providers, viewing test results, and making medical appointments online)

Exclusion criteria:

- “Not Reported” responses on any of the three outcome variables
- Respondents who reported not needing medical care in the last 12 months to avoid ambiguity in interpreting the delayed-care measure
- Respondents with unreported Internet health-information-seeking status

### 2.3. Study Variables

The outcome variables, Internet use to message healthcare providers, Internet use to view test results, and Internet use to make appointments, were treated as binary variables: “In the past 12 months, have you used the Internet to…” Responses were recoded as 1 = Yes and 0 = No. Five main independent variables were used across all three models. Internet use for health information was assessed with the question, “In the past 12 months, have you used the Internet to look for health or medical information?” and coded as 0 = No and 1 = Yes. Social media use for health decision-making was assessed with the statement, “I use information from social media to make decisions about my health.” Responses were coded as Strongly Agree, Somewhat Agree, Somewhat Disagree, or Strongly Disagree, with “Strongly Agree” serving as the reference category. Confidence in finding helpful health resources on the Internet was assessed with the question, “How confident are you that you can find helpful health resources on the Internet?” Responses were categorized as completely confident, very confident, somewhat confident, a little confident, and not confident at all. “Completely confident” served as the reference category in the multivariable regression analyses. Missing or inapplicable responses were retained in the “Not Reported” category.

The other two independent variables were trust in the healthcare system and delay in needed care. Trust in the healthcare system was assessed with the question, “How much do you trust the healthcare system (for example, hospitals, pharmacies, and other organizations involved in healthcare)?” Responses were categorized as very, somewhat, a little, and not at all, with “Very” serving as the reference category. Delayed needed care was assessed with the question, “In the past 12 months, did you delay or not get medical care you felt you needed—such as seeing a doctor, a specialist, or other health professional?” Responses were categorized as Yes or No, with “No” serving as the reference category. As described above, respondents who indicated that they did not need medical care during the previous 12 months were excluded from the regression analytic sample. Control variables included chronic condition status, biological sex, education, age, and race/ethnicity. Chronic condition status was derived from self-reported medical conditions and categorized as Yes or No, with “No” serving as the reference category.

### 2.4. Statistical Analyses

Descriptive statistics were used to summarize respondent characteristics and study variables. Three separate multivariable logistic regression models were estimated to examine factors associated with (1) using the Internet to message a healthcare provider or provider’s office, (2) viewing medical test results online, and (3) making medical appointments online. All models included Internet use for health information, social media use for health decision-making, confidence in finding helpful health resources online, trust in the healthcare system, delayed needed care, chronic condition status, biological sex, education, age, and race/ethnicity. Results are reported as adjusted odds ratios (AORs) with 95% confidence intervals (CIs). Statistical significance was assessed at *p* < 0.05. All analyses incorporated HINTS 6 survey weights to account for the complex sampling design and produce nationally representative estimates. The full-sample weight was used for population estimates, and the 50 replicate weights provided with HINTS 6 were used for variance estimation using the delete-one jackknife (JK1) replication method. Missing or inapplicable responses were retained as a “Not Reported” category and entered as categorical. Analyses were conducted using Stata version 18.0. The study was determined to be exempt by the Georgia Southern University Institutional Review Board (protocol H25121; 8 November 2024).

## 3. Results

**Figure 1:**
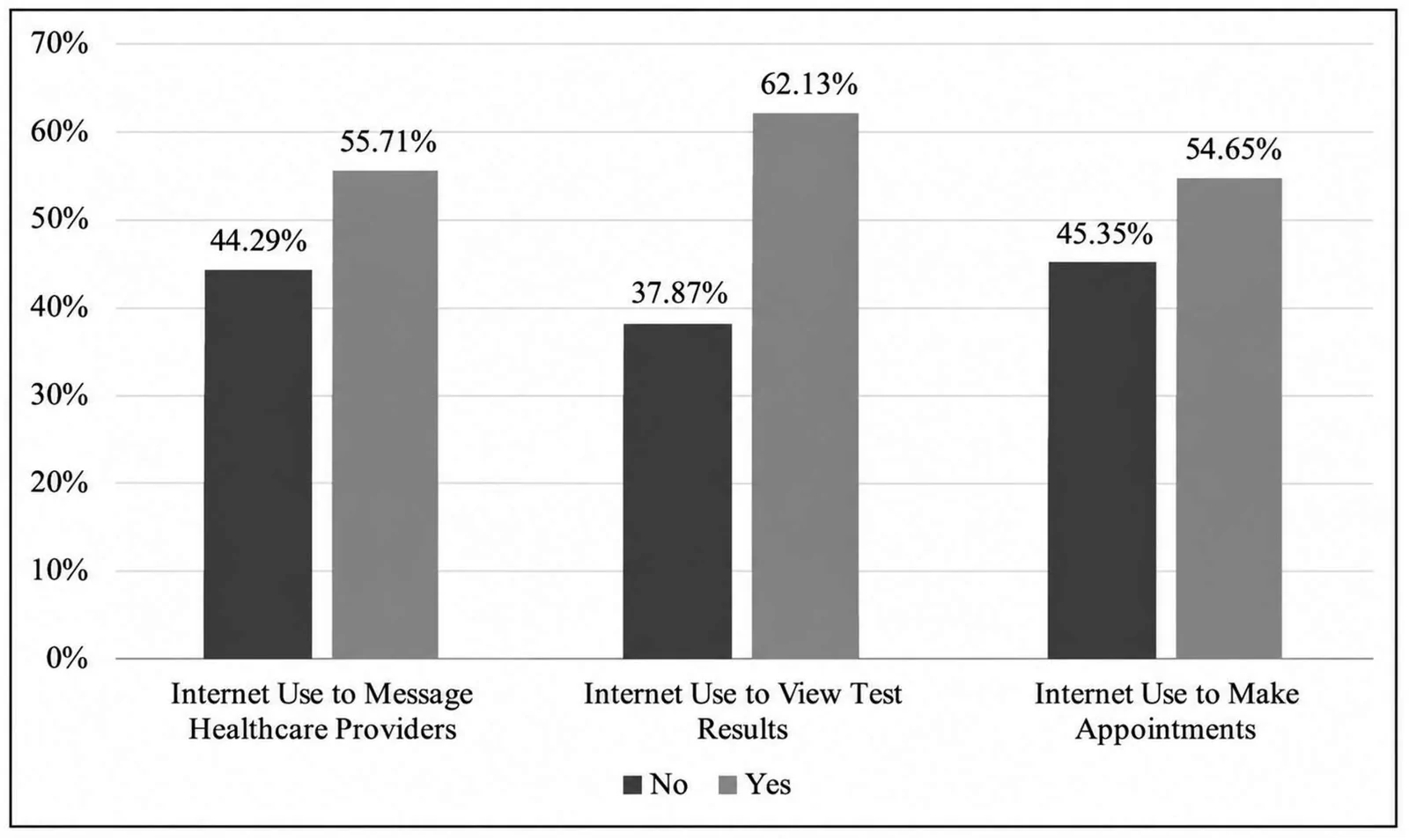
Descriptive Statistics of Digital Health Service Engagement.

Descriptive analyses included 5983 respondents with valid responses for the three digital health engagement outcomes. The multivariable regression analyses included 4675 respondents after applying the additional exclusions described in the Methods. For the three digital health engagement outcomes, 55.71% reported using the Internet to message a healthcare provider or provider’s office, 62.13% reported viewing medical test results online, and 54.65% reported making medical appointments online (Figure 1).

Descriptive statistics for the study variables are presented in Table 1. Among respondents in the descriptive sample, 56.16% were female, and the largest age group was 50–64 years (28.50%). Most respondents reported using the Internet to look for health or medical information (72.79%). In contrast, relatively few strongly agreed that they used information from social media to make decisions about their health (0.94%), while 66.97% strongly disagreed. Confidence in finding helpful health resources on the Internet varied, with 11.73% reporting that they were completely confident, 33.70% very confident, and 38.41% somewhat confident. Most respondents reported trusting the healthcare system either somewhat (45.96%) or very much (37.34%). Among the descriptive sample, 59.62% reported that they had not delayed needed care, while 29.22% reported that they had delayed needed care; 11.16% were classified as “Not Reported” for this measure.

Three separate multivariable logistic regression models examined factors associated with (1) using the Internet to message a healthcare provider or provider’s office, (2) viewing medical test results online, and (3) making medical appointments online. Across all three models, Internet health-information seeking was significantly associated with higher odds of digital health engagement.

### 3.1. Model 1

Table 2 presents the multivariable logistic regression results for factors associated with using the Internet to message healthcare providers or their offices. Internet health-information seeking was strongly associated with higher odds of messaging a healthcare provider (AOR = 9.88, 95% CI [7.59, 12.87], *p* < 0.001). Respondents who somewhat disagreed that they used social media information to make health decisions also had higher odds of messaging a provider than those who strongly agreed (AOR = 4.49, 95% CI [1.10, 18.37], *p* = 0.037); however, this estimate should be interpreted cautiously because of the small cell sizes for the social media categories.

Compared with respondents who were completely confident in their ability to find helpful health resources online, those who were somewhat confident (AOR = 0.63, 95% CI [0.41, 0.97], *p* = 0.038), a little confident (AOR = 0.52, 95% CI [0.31, 0.88], *p* = 0.016), or not confident at all (AOR = 0.25, 95% CI [0.14, 0.45], *p* < 0.001) had lower odds of messaging a healthcare provider. Respondents with chronic conditions had higher odds of messaging a provider than those without chronic conditions (AOR = 1.41, 95% CI [1.10, 1.80], *p* = 0.008). Non-Hispanic Black respondents had lower odds than non-Hispanic White respondents (AOR = 0.72, 95% CI [0.53, 0.97], *p* = 0.031).

### 3.2. Model 2

Table 3 presents the multivariable logistic regression results for factors associated with viewing medical test results online. Internet health-information seeking was strongly associated with higher odds of viewing test results online (AOR = 14.46, 95% CI [10.80, 19.37], *p* < 0.001). Compared with respondents who were completely confident in their ability to find helpful health resources online, those who were not confident at all had lower odds of viewing test results (AOR = 0.27, 95% CI [0.16, 0.45], *p* < 0.001). Respondents who reported delayed needed care also had lower odds of viewing test results than those who did not delay needed care (AOR = 0.67, 95% CI [0.48, 0.94], *p* = 0.023).

Respondents with a college degree or higher had higher odds of viewing test results online than those with less than a high school education (AOR = 2.72, 95% CI [1.24, 5.95], *p* = 0.013). Non-Hispanic Black respondents had lower odds of viewing test results online than non-Hispanic White respondents (AOR = 0.61, 95% CI [0.42, 0.90], *p* = 0.013).

### 3.3. Model 3

Table 4 presents the multivariable logistic regression results for factors associated with making medical appointments online. Internet health-information seeking was strongly associated with higher odds of making appointments online (AOR = 7.38, 95% CI [5.16, 10.54], *p* < 0.001). Compared with respondents who were completely confident in their ability to find helpful health resources online, those who were somewhat confident (AOR = 0.47, 95% CI [0.31, 0.71], *p* = 0.001), a little confident (AOR = 0.39, 95% CI [0.23, 0.65], *p* = 0.001), or not confident at all (AOR = 0.16, 95% CI [0.09, 0.28], *p* < 0.001) had lower odds of making medical appointments online.

Age was also associated with online appointment-making. Compared with respondents aged 18–34 years, those aged 35–49 years (AOR = 0.64, 95% CI [0.45, 0.91], *p* = 0.014), 50–64 years (AOR = 0.52, 95% CI [0.38, 0.72], *p* < 0.001), 65–74 years (AOR = 0.33, 95% CI [0.23, 0.48], *p* < 0.001), and 75 years or older (AOR = 0.37, 95% CI [0.25, 0.54], *p* < 0.001) had lower odds of making medical appointments online.

## 4. Discussion

This study examined factors associated with the use of online patient health services among U.S. adults. These services included using the Internet to message healthcare providers or their offices, view medical test results, and make medical appointments. Across all three models, Internet use to look for health information had a strong and consistent association with digital health engagement. Respondents who used the Internet to look for health information had higher odds of using each of the three online health services examined in this study. Internet use for health information may reflect greater prior experience of online digital health tools among healthcare consumers, including familiarity and comfort with finding and using health resources online. This may explain why individuals who already use the Internet for health information are also more likely to use it for other healthcare needs [11]. The strong association between Internet health-information seeking and all three forms of digital health engagement should also be interpreted with some caution. Health-information seeking and the three outcomes are conceptually related Internet-based health behaviors and may, in part, reflect a broader propensity or familiarity with using the Internet for health-related purposes. Therefore, these associations should not be interpreted as evidence that health-information seeking itself leads to greater use of online healthcare services. In this context, UTAUT provides a theory-informed framework for considering factors associated with digital engagement rather than a model formally tested by the HINTS 6 measures. The increasing availability of health information online has also provided individuals with more opportunities to learn about health conditions and participate in decisions about their health [13].

The association between social media use for health decision-making and digital health engagement was less consistent. Social media use was significantly associated with messaging healthcare providers but was not significantly associated with viewing test results or making medical appointments. Respondents who somewhat disagreed that they used social media information to make health decisions had higher odds of messaging healthcare providers than those who strongly agreed. One possible explanation is that some individuals may use provider messaging to clarify health information encountered through social media [14]. Concerns about misinformation, contradictory information, and information overload on social media may also lead some individuals to seek information directly from healthcare providers [15,16]. However, the HINTS 6 data do not indicate what information respondents encountered on social media or why they contacted their providers. Therefore, this finding should be interpreted cautiously. Overall, social media use was not consistently associated with the three forms of digital health engagement examined in this study.

Confidence in finding helpful health resources on the Internet was also associated with digital health engagement. Compared with respondents who were completely confident, respondents with lower levels of confidence generally had lower odds of using the online health services examined. This pattern was particularly evident for messaging healthcare providers and making appointments online. Individuals who have less confidence in finding helpful health information may also be less comfortable navigating other online health services [13]. This finding is relevant to the digital divide because access to technology alone may not ensure effective use. Individuals also need the confidence and ability to navigate digital resources [17]. As more healthcare services become available online, differences in digital confidence may affect how patients use these services.

Trust in the healthcare system was not consistently associated with the three digital health engagement outcomes. This suggests that general trust in the healthcare system may not, by itself, explain whether individuals use the Internet to message providers, view test results, or make appointments. Trust remains an important part of the patient–provider relationship, but the decision to use a particular digital health service may involve other factors, including access, digital confidence, previous experience with technology, and whether the service is available through the patient’s healthcare organization [18]. Future research may benefit from examining trust in specific digital health services or technologies rather than general trust in the healthcare system.

Delayed needed care was associated with lower odds of viewing medical test results online but was not significantly associated with messaging healthcare providers or making appointments. Individuals who experience delays in needed care may also be less engaged with some aspects of healthcare follow-up, although the cross-sectional design of this study does not establish the direction of this relationship. Previous research has identified several reasons for delaying or avoiding medical care, including fear and uncertainty about potential diagnoses [19,20]. Additional research is needed to understand why delayed needed care was associated with viewing test results but not the other digital health services examined in this study.

Respondents with chronic conditions had higher odds of using the Internet to message healthcare providers compared with respondents without chronic conditions. Individuals managing chronic conditions may have more frequent healthcare needs and may use online messaging to maintain communication with their healthcare providers [21]. Online communication may be particularly useful when patients have questions about ongoing care, medications, or management of their conditions. However, HINTS 6 does not provide sufficient information in this analysis about the severity or duration of respondents’ chronic conditions. Future studies could examine whether the frequency and type of online communication differ according to the number, type, or severity of chronic conditions.

Some demographic differences were also observed. Non-Hispanic Black respondents had lower odds of messaging healthcare providers and viewing medical test results online compared with non-Hispanic White respondents. Age differences were most evident for making medical appointments online. Compared with adults aged 18–34 years, respondents in the older age groups had lower odds of making appointments online. These differences may not be explained solely by individual digital skills. Some patients may prefer to schedule appointments by telephone, while others may receive care from healthcare organizations that do not offer the same online scheduling options [22]. Similarly, differences in access to patient portals, Internet connectivity, digital literacy, and healthcare-system practices may contribute to differences in the use of online health services. These findings suggest that both individual and healthcare-system factors should be considered when examining differences in digital health engagement. More broadly, digital engagement should not be viewed solely as an individual capacity or form of digital readiness. Opportunities to use and benefit from online healthcare services may also depend on access to reliable connectivity and patient portals, digital literacy, portal usability, and healthcare-system practices. Consequently, efforts to promote equitable digital engagement may need to address both individual-level barriers and the broader organizational and social conditions that shape access to and use of digital healthcare services.

The findings have implications for healthcare organizations as they continue to expand digital services. Providing an online service does not necessarily mean that all patients will use it. Patients may differ in their confidence, experience with technology, and ability to find and use online health resources. Healthcare organizations can help by making patient portals and other digital services easier to navigate and by providing clear instructions or assistance to patients who need additional support. At the same time, maintaining alternative ways to communicate with providers and schedule care may be important for patients who are less comfortable using digital services. Efforts to increase digital engagement should consider these differences so that expanded use of technology does not contribute to existing disparities in access to healthcare services.

### 4.1. Limitations of the Study

This study has several limitations. First, the cross-sectional design of HINTS 6 does not allow the temporal order or causality of the observed associations to be established. Longitudinal research would help determine how Internet health-information seeking and engagement with online health services change over time. Second, the study relied on self-reported data, which may be affected by recall or social desirability bias. Future studies could incorporate objective measures, such as electronic health record or patient portal usage data, to examine how often patients message providers, view test results, and make appointments online. Third, the term “Internet” can include different types of digital activities and platforms. The measures used in this study do not capture all of these activities. Fourth, the social media measure does not identify the specific platforms used, the type of health information encountered, or the frequency of exposure. These differences may be important when examining how social media relates to healthcare decisions and digital health engagement. Fifth, the strong association between using the Internet to search for health information and each of the three digital engagement outcomes should be interpreted with caution. Across all three models, adjusted odds ratios were large (7.38 to 14.46), but these measures may not represent fully distinct constructs. Internet health information seeking and digital engagement may both reflect a broader underlying propensity or familiarity with using the Internet for health-related purposes. Patients who already look up symptoms or navigate health resources online may be more likely to use patient portals, telehealth, or other digital health tools, in part because these behaviors draw on the same experience. Accordingly, this association should not be interpreted as evidence that information seeking leads to digital engagement. In addition, measures of access or use do not fully capture barriers such as low digital health literacy and mistrust of online health information, which may limit the extent to which the current models isolate their influence. Future studies that directly measure constructs such as digital health literacy and self-efficacy, ideally with longitudinal designs, would help clarify how these behaviors relate to one another. Finally, although survey weights were used to produce nationally representative estimates, the findings reflect the measures available in HINTS 6 and may not capture other factors that influence the use of digital health services. The timing of data collection should also be considered when interpreting the findings. HINTS 6 data were collected in 2022, and digital healthcare technologies, patient portal functionality, and patterns of technology use continue to evolve rapidly. The findings therefore characterize associations during the period represented by HINTS 6 and should not be assumed to reflect current patterns without further study.

### 4.2. Recommendations for Further Research

Future research should examine whether these associations persist in more recent data and should incorporate more direct measures of digital literacy, access to patient portals, connectivity, usability, and organizational support for digital healthcare. Longitudinal studies may also help clarify the temporal relationships between health-information seeking and subsequent engagement with online healthcare services.

## 5. Conclusions

This study provides insight into the factors associated with engagement with online patient health services. Internet use for health information was strongly associated with messaging healthcare providers, viewing test results, and making appointments online. Confidence in finding helpful health resources online was also important, with lower confidence associated with lower odds of engaging in online patient health services. Social media use was associated with messaging providers but not with viewing test results or making appointments, while delayed needed care was associated with lower odds of viewing test results. Demographic differences were also observed, particularly by age and race/ethnicity. As healthcare continues to become more digital, these findings may help healthcare organizations identify opportunities to improve patient engagement and address disparities in the use of digital health services.

The findings have several implications for digital health engagement and the use of online patient health services. Offering online health services does not guarantee that patients will use them, and lower confidence in finding helpful health resources online was associated with lower odds of engagement. Stakeholders such as health organizations and policymakers may therefore benefit from investing in patient portals with improved user experience and from providing guidance that supports patient confidence. Another implication is that the results showed lower odds of scheduling appointments online among older adults, and of messaging providers and viewing test results among non-Hispanic Black respondents. This suggests a risk of widening existing disparities if the expansion of digital health services does not attend to these differences. Moreover, system- and patient-level barriers should be addressed in efforts to increase engagement with digital health tools. It is also important to maintain non-digital options for patients who prefer or require them.

## Figures and Tables

**Table 1:** Descriptive statistics for the variables in the study.

Measures	Frequency (Unweighted)	Percent (% Weighted)
**Internet Use for Health Information**		
No	1628	27.21
Yes	4355	72.79
**Social Media to Make Decisions**		
Strongly Agree	56	0.94
Somewhat Agree	728	12.17
Somewhat Disagree	1015	16.96
Strongly Disagree	4007	66.97
Not Reported	177	2.96
**Confidence in Internet Health Resources**		
Completely Confident	702	11.73
Very Confident	2016	33.70
Somewhat Confident	2298	38.41
A little Confident	551	9.21
Not Confident at All	316	5.28
Not Reported	100	1.67
**Trust in HCSystem**		
Very	2234	37.34
Somewhat	2750	45.96
A little	699	11.68
Not at all	174	2.91
Not Reported	126	2.11
**Delay of Needed Care**		
No, I did not delay care I felt I needed	3567	59.62
Yes, I delayed care I felt I needed	1748	29.22
Not Reported	668	11.16
**Chronic Conditions**		
No	1816	30.35
Yes	3981	66.54
Not Reported	186	3.11
**Biological Sex**		
Male	2231	37.29
Female	3360	56.16
Not Reported	392	6.55
**Education**		
Less than high school	323	5.40
High school graduate	988	16.51
Some college	1608	26.88
College graduate or more	2676	44.73
Not Reported	388	6.49
**Age**		
18–34	938	15.68
35–49	1221	20.41
50–64	1705	28.50
65–74	1278	21.36
75 and above	764	12.77
Not Reported	77	1.29
**Race/Ethnicity**		
Non-Hispanic white	3120	52.15
Non-Hispanic Black or African American	849	14.19
Hispanic	938	15.68
Other	463	7.74
Not Reported	613	10.25
*Note*: *N* = 5983.		

**Table 2:** Multivariable logistic regression of use of the Internet to message healthcare providers and providers’ offices.

95% CI				
Variable	AOR	Lower	Upper	*p*-Value
**Internet Use for Health Information**				
Yes	9.88	7.59	12.87	***<0.001***
No	…	…	…	
**Social Media to Make Decisions**				
Not Reported	1.64	0.34	7.98	0.534
Strongly disagree	4.08	0.97	17.12	0.055
Somewhat disagree	4.49	1.10	18.37	***0.037***
Somewhat agree	3.00	0.63	14.36	0.164
Strongly agree	…	…	…	
**Confident Internet Health**				
Not Reported	0.57	0.23	1.42	0.220
Not confident at all	0.25	0.14	0.45	***<0.001***
A little confident	0.52	0.31	0.88	***0.016***
Somewhat confident	0.63	0.41	0.97	***0.038***
Very confident	1.17	0.79	1.72	0.433
Completely confident	…	…	…	
**Trust in HC System**				
Not Reported	3.68	1.26	10.73	***0.018***
Not at all	1.42	0.60	3.39	0.422
A little	0.79	0.52	1.21	0.274
Somewhat	0.88	0.69	1.13	0.320
Very	…	…	…	
**Delay in Needed Care**				
Not Reported	0.31	0.20	0.46	***<*** ***0*** ***.001***
Yes	1.12	0.89	1.40	0.320
No	…	…	…	
**Chronic Conditions**				
Not Reported	2.10	0.95	4.63	0.066
Yes	1.41	1.10	1.80	***0.008***
No	…	…	…	
**Biological Sex**				
Not Reported	2.99	0.26	33.93	0.370
Female	1.08	0.88	1.34	0.441
Male	…	…	…	
**Education**				
Not Reported	0.54	0.02	13.61	0.705
College graduate or more	1.55	0.76	3.14	0.223
Some college	1.24	0.61	2.54	0.542
High school graduate	0.57	0.29	1.14	0.110
Less than high school	…	…	…	
**Age**				
Not Reported	0.56	0.21	1.48	0.235
35–49	1.39	1.00	1.95	0.050
50–64	1.26	0.87	1.83	0.215
65–74	0.82	0.57	1.18	0.276
75 and above	0.70	0.46	1.07	0.102
18–34	…	…	…	
**Race/Ethnicity**				
Not Reported	0.60	0.31	1.14	0.116
Non-Hispanic Black or African American	0.72	0.53	0.97	***0.031***
Hispanic	0.78	0.56	1.09	0.141
Other	1.04	0.73	1.50	0.816
Non-Hispanic white	…	…	…	

Note: *p* < 0.05; *p*-values are highlighted in italics and bolded where statistically significant; the estimates for social media categories may be unstable due to small cell sizes. Estimates were obtained using survey-weighted multivariable logistic regression. The HINTS 6 full-sample weight was used for population estimates, and the 50 replicate weights were used for variance estimation using the delete-one jackknife (JK1) method. Abbreviations: AOR, adjusted odds ratio; CI, confidence interval. The … represent a reference category.

**Table 3:** Multivariable logistic regression of the use of the Internet to view their test results.

95% CI				
Variable	AOR	Lower	Upper	*p*-Value
**Internet Use for Health Information**				
Yes	14.46	10.80	19.37	***<*** ***0*** ***.001***
No	…	…	…	
**Social Media to Make Decisions**				
Not Reported	0.32	0.04	2.34	0.256
Strongly disagree	0.58	0.09	3.87	0.568
Somewhat disagree	0.52	0.08	3.35	0.482
Somewhat agree	0.39	0.06	2.45	0.306
Strongly agree	…	…	…	
**Confident Internet Health**				
Not Reported	0.28	0.08	1.00	0.050
Not confident at all	0.27	0.16	0.45	***<*** ***0*** ***.001***
A little confident	0.61	0.33	1.11	0.104
Somewhat confident	0.73	0.43	1.24	0.242
Very confident	1.09	0.63	1.89	0.757
Completely confident	…	…	…	
**Trust in HCSystem**				
Not Reported	2.93	1.16	7.44	***0.024***
Not at all	0.99	0.38	2.55	0.985
A little	0.76	0.52	1.11	0.150
Somewhat	0.99	0.74	1.31	0.933
Very	…	…	…	
**Delay in Needed Care**				
Not Reported	0.31	0.20	0.49	***<*** ***0*** ***.001***
Yes	0.67	0.48	0.94	***0.023***
No	…	…	…	
**Chronic Conditions**				
Not Reported	0.86	0.35	2.10	0.740
Yes	1.12	0.86	1.47	0.383
No	…	…	…	
**Biological Sex**				
Not Reported	0.58	0.17	1.99	0.380
Female	1.27	0.98	1.64	0.068
Male	…	…	…	
**Education**				
Not Reported	7.60	1.61	35.96	***0.012***
College graduate or more	2.72	1.24	5.95	***0.013***
Some college	1.89	0.89	4.02	0.095
High school graduate	0.95	0.42	2.13	0.896
Less than high school	…	…	…	
**Age**				
Not Reported	0.71	0.27	1.91	0.495
35–49	1.16	0.76	1.75	0.490
50–64	1.13	0.72	1.78	0.583
65–74	0.75	0.48	1.16	0.194
75 and above	0.66	0.42	1.05	0.078
18–34	…	…	…	
**Race/Ethnicity**				
Not Reported	0.79	0.45	1.40	0.411
Non-Hispanic Black or African American	0.61	0.42	0.90	***0.013***
Hispanic	1.08	0.77	1.53	0.644
Other	1.25	0.74	2.11	0.391
Non-Hispanic white	…	…	…	

Note: The italics and bold formatting is used to identify statistically significant *p*-values (*p* < 0.05). The … represent a reference category. Estimates were obtained using survey-weighted multivariable logistic regression. The HINTS 6 full-sample weight was used for population estimates, and the 50 replicate weights were used for variance estimation using the delete-one jackknife (JK1) method. Abbreviations: AOR, adjusted odds ratio; CI, confidence interval.

**Table 4:** Multivariable logistic regression of use of the Internet to make medical appointments.

	95% CI				
	Variable	AOR	Lower	Upper	*p*-Value
	**Internet Use for Health Information**				
	Yes	7.38	5.16	10.54	***<*** ***0*** ***.001***
	No	…	…	…	
	**Social Media to Make Decisions**				
	Not Reported	0.19	0.03	1.10	0.064
	Strongly disagree	0.60	0.12	2.92	0.518
	Somewhat disagree	0.63	0.13	3.01	0.551
	Somewhat agree	0.60	0.13	2.83	0.508
	Strongly agree	…	…	…	
	**Confident Internet Health**				
	Not Reported	0.28	0.13	0.58	***0.001***
Not confident at all		0.16	0.09	0.28	***<0.001***
A little confident		0.39	0.23	0.65	***0.001***
Somewhat confident		0.47	0.31	0.71	***0.001***
	Very confident	0.68	0.44	1.05	0.080
	Completely confident	…	…	…	
	**Trust in HCSystem**				
	Not Reported	1.86	0.67	5.17	0.226
	Not at all	0.76	0.34	1.73	0.508
	A little	0.94	0.64	1.36	0.723
	Somewhat	0.85	0.68	1.06	0.138
	Very	…	…	…	
	**Delay in Needed Care**				
	Not Reported	0.38	0.24	0.61	***<*** ***0*** ***.001***
	Yes	0.95	0.74	1.22	0.673
	No	…	…	…	
	**Chronic Conditions**				
	Not Reported	2.20	0.96	5.03	0.062
	Yes	0.99	0.76	1.28	0.922
	No	…	…	…	
	**Biological Sex**				
	Not Reported	0.57	0.15	2.20	0.403
	Female	0.95	0.75	1.20	0.685
	Male	…	…	…	
	**Education**				
	Not Reported	2.67	0.59	12.10	0.197
	College graduate or more	1.60	0.87	2.97	0.131
	Some college	1.30	0.68	2.49	0.420
	High school graduate	0.68	0.34	1.35	0.264
	Less than high school	…	…	…	
	**Age**				
	Not Reported	0.55	0.23	1.33	0.179
	35–49	0.64	0.45	0.91	***0.014***
	50–64	0.52	0.38	0.72	***<0.001***
	65–74	0.33	0.23	0.48	***<0.001***
	75 and above	0.37	0.25	0.54	***<0.001***
	18–34	…	…	…	
	**Race/Ethnicity**				
	Not Reported	0.78	0.45	1.35	0.368
	Non-Hispanic Black or African American	1.28	0.91	1.79	0.154
	Hispanic	1.25	0.90	1.73	0.183
	Other	1.12	0.71	1.78	0.623
	Non-Hispanic white	…	…	…	

Note: The italics and bold formatting is used to identify statistically significant *p*-values (*p* < 0.05). Estimates were obtained using survey-weighted multivariable logistic regression. The HINTS 6 full-sample weight was used for population estimates, and the 50 replicate weights were used for variance estimation using the delete-one jackknife (JK1) method. Abbreviations: AOR, adjusted odds ratio; CI, confidence interval. The … represent a reference category.

## Data Availability

The original data presented in the study are openly available from the Health Information National Trends Survey (HINTS) data repository at https://hints.cancer.gov/data/Default.aspx (Accessed on 18 January 2025).

## Abbreviations

The following abbreviations are used in this manuscript:

AOR	Adjusted Odds Ratio
CI	Confidence Interval
HINTS	Health Information National Trends Survey
IRB	Institutional Review Board
JK1	Delete-One Jackknife
UTAUT	Unified Theory of Acceptance and Use of Technology
